# Antimicrobial resistance in *Escherichia coli* and *Pseudomonas aeruginosa* before and after the coronavirus disease 2019 (COVID-19) pandemic in the Dominican Republic

**DOI:** 10.1017/ash.2022.347

**Published:** 2022-12-06

**Authors:** Alfredo J. Mena Lora, Chrystiam Sorondo, Belkis Billini, Patricia Gonzalez, Susan C. Bleasdale

**Affiliations:** 1 University of Illinois at Chicago, Chicago, Illinois, United States; 2 Amadita Laboratories, Santo Domingo, Dominican Republic

## Abstract

**Objective:**

To describe antimicrobial resistance before and after the COVID-19 pandemic in the Dominican Republic.

**Design:**

Retrospective study.

**Setting:**

The study included 49 outpatient laboratory sites located in 13 cities nationwide.

**Participants:**

Patients seeking ambulatory microbiology testing for urine and bodily fluids

**Methods:**

We reviewed antimicrobial susceptibility reports for *Escherichia coli* isolates from urine and *Pseudomonas aeruginosa* (PSAR) from bodily fluids between January 1, 2018, to December 31, 2021, from deidentified susceptibility data extracted from final culture results.

**Results:**

In total, 27,718 urine cultures with *E. coli* and 2,111 bodily fluid cultures with PSAR were included in the analysis. On average, resistance to ceftriaxone was present in 25.19% of *E. coli* isolated from urine each year. The carbapenem resistance rates were 0.15% for *E. coli* and 3.08% for PSAR annually. The average rates of *E. coli* with phenotypic resistance consistent with possible extended-spectrum β-lactamase (ESBL) in urine were 25.63% and 24.75%, respectively, before and after the COVID-19 pandemic. The carbapenem resistance rates in urine were 0.11% and 0.20%, respectively, a 200% increase. The average rates of PSAR with carbapenem resistance in bodily fluid were 2.33% and 3.84% before and after the COVID-19 pandemic, respectively, a 130% percent increase.

**Conclusions:**

Resistance to carbapenems in PSAR and *E. coli* after the COVID-19 pandemic is rising. These resistance patterns suggest that ESBL is common in the Dominican Republic. Carbapenem resistance was uncommon but increased after the COVID-19 pandemic.

Antimicrobial resistance (AMR) is a major global threat.^1^ Low- and middle-income countries (LMICs) have unique challenges that may contribute to AMR.^2^ In many LMICs, antimicrobials can be purchased without prescriptions and are widely available.^3–6^ These factors may be driving an increase in antimicrobial use (AU) and AMR in LMICs over the past decade.^7^ The COVID-19 pandemic has further compounded this challenge, increasing AU in both inpatient and outpatient settings.^8–11^

Developing antimicrobial stewardship programs (ASPs) in LMICs is critical to help optimize antimicrobial use and curb AMR. Understanding local susceptibility patterns is a key first step toward the development of treatment guidelines for ASPs. The lack of local susceptibility data to guide therapy could lead to spiraling empiricism, furthering antimicrobial pressure and worsening AMR. Data are lacking on local susceptibility in the Dominican Republic and the impact of the COVID-19 pandemic on AMR. We sought to describe resistance in the Dominican Republic and the impact of the COVID-19 pandemic.

## Methods

We performed a retrospective review of antimicrobial susceptibility reports for *Escherichia coli* and *Pseudomonas aeruginosa* (PSAR) isolates from an outpatient clinical laboratory in the Dominican Republic. A report was developed for *E. coli* isolates from urine and PSAR isolates from bodily fluids processed between January 1, 2018, and December 31, 2021. Isolates processed between January 1, 2018, and December 31, 2019, were considered prepandemic isolates. Isolates processed between January 1, 2020, and December 31, 2021, were considered postpandemic isolates. These 2 groups reflect the annual antibiograms before and after COVID-19. The bodily fluid category represented samples from any abscess, body cavity, or anatomical site. The report included adults and children and was generated from deidentified susceptibility data extracted from all final culture results during the study period. The University of Illinois at Chicago Institutional Review Board approved this study.

### Study site

**Fig. 1.**
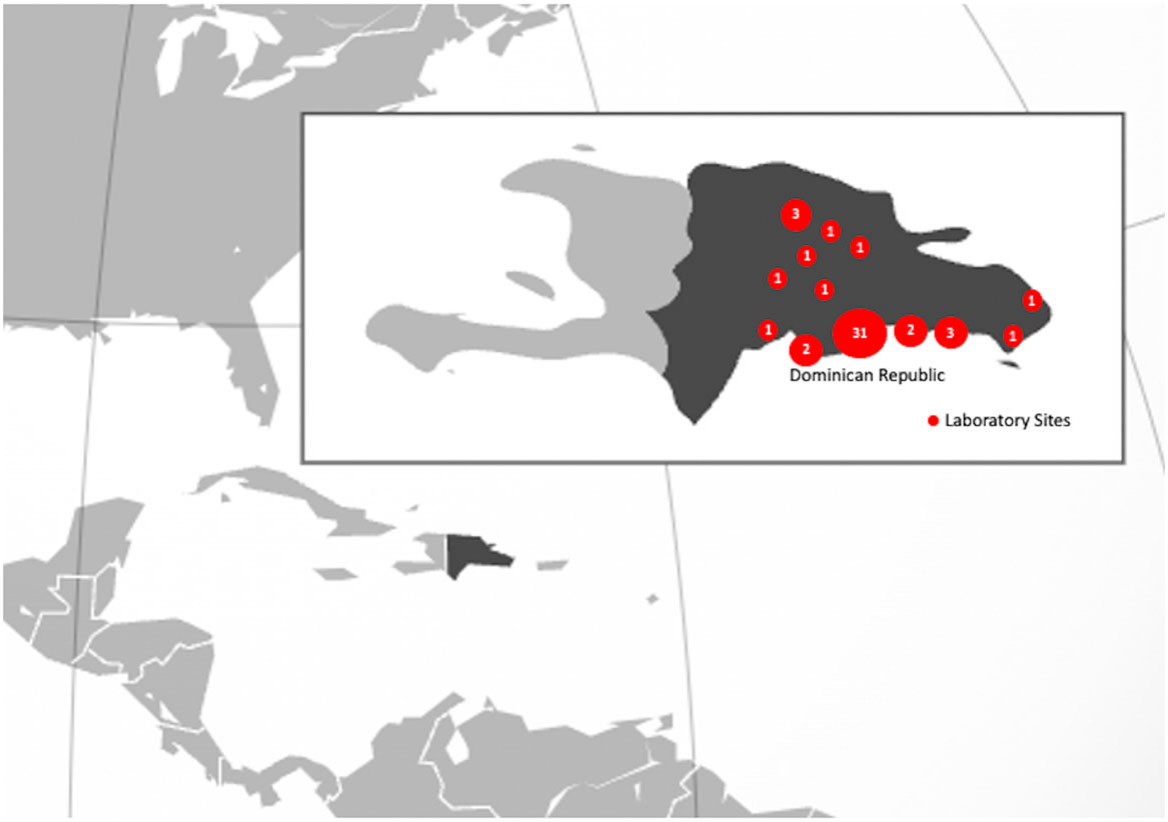
Locations of the Amadita Laboratory outpatient collection sites across the Dominican Republic.

Amadita Laboratories is a private commercial laboratory company that provides outpatient services nationwide in the Dominican Republic. There are 49 outpatient laboratory sites across the Dominican Republic, representing 13 urban areas and all geographic regions of the country (Fig. 1).

### Laboratory workflow and microbiology testing

Samples are collected at the outpatient laboratory sites and are processed centrally in the city of Santo Domingo. The central laboratory is equipped with the VITEK MS, VITEK 2 XL, VITEK 2 Compact (bioMèrieux, Marcy-l'Étoile, France) and advanced expert systems (AESs) for initial analysis. The VITEK 2 XL and VITEK 2 Compact are used for the initial testing. VITEK 2 XL uses the AST-GN67, AST XN05, and AST XN08 susceptibility cards, and VITEK 2 Compat uses the AST-GN67, AST XN05, and AST XN08 cards. VITEK MS is used for matrix-assisted laser desorption/ionization time-of-flight mass spectroscopy (MALDI-TOF MS) analysis of organisms. AES software versions 8.0 and 9.2 were used for analysis and interpretation of the antibiogram. Additional confirmatory testing was performed using the Kirby-Bauer method via the MASTDISCS Combi AMP-C, ESBL ID MAST GROUP, and MASTDISCS Combi CARBA PLUS discs (Mast Group, Bootle, UK). Sensitivity discs for individual carbapenems and the E-test for meropenem were used to confirm resistance.

For *E. coli*, a minimum inhibitory concentration (MIC) of ≥4 to ceftriaxone was considered resistant and possible extended-spectrum β-lactamase (ESBL) whereas an MIC of ≥8 μg/mL to meropenem was considered resistant (carbapenem-resistant Enterobacterales or CRE). For PSAR, an MIC of ≥32 μg/mL to ceftazidime was considered resistant and possible ESBL while an MIC of ≥8 μg/mL for meropenem was considered resistant (carbapenem-resistant *Pseudomonas auriginosa* or CR-PSAR). Thresholds for resistance were based on Clinical and Laboratory Standards Institute (CLSI) M100.

## Results

**Fig. 2.**
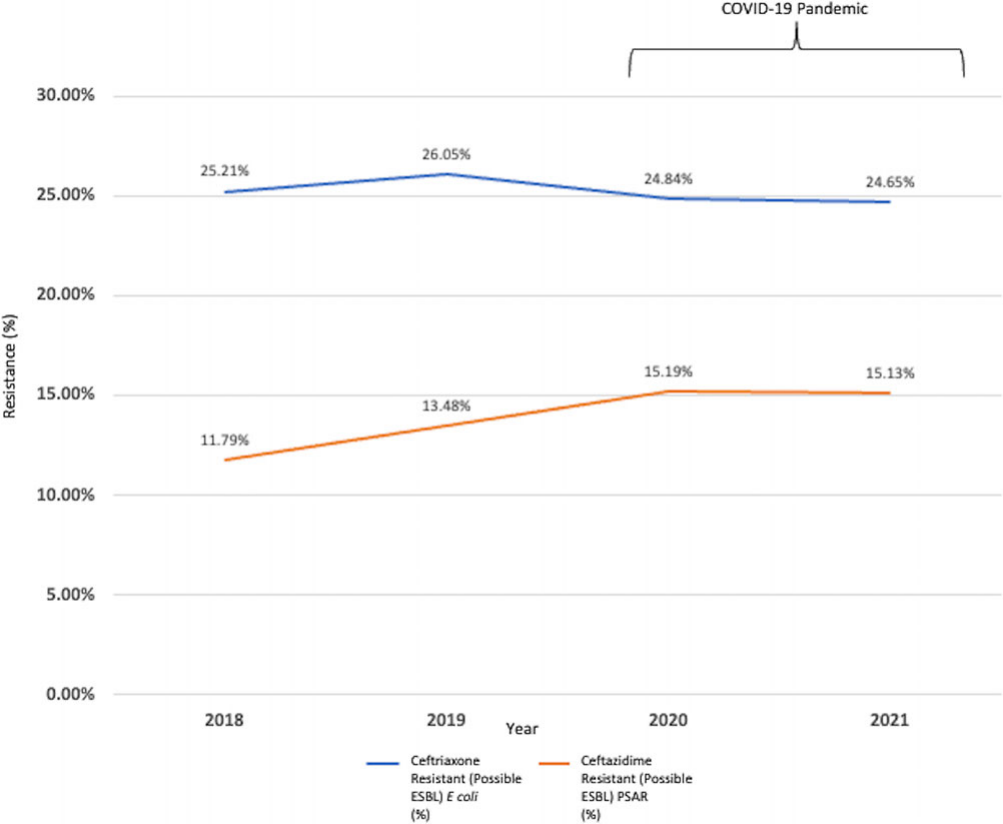
*Escherichia coli* from urine with ceftriaxone resistance and *Pseudomonas aeruginosa* (PSAR) from bodily fluids with ceftazidime resistance (possible extended-spectrum β-lactamase or ESBL) before and after the COVID-19 pandemic.

**Fig. 3.**
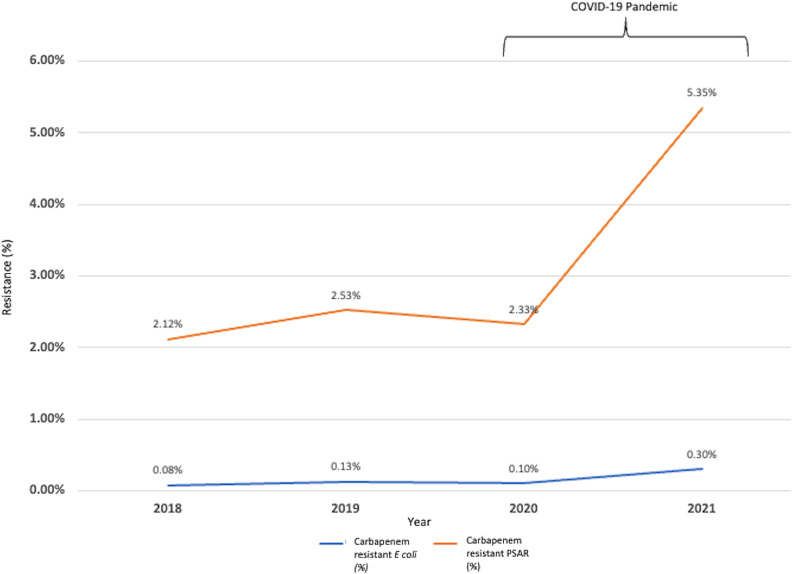
*Escherichia coli* from urine and *Pseudomonas aeruginosa* (PSAR) from bodily fluids with carbapenem resistance before and after the COVID-19 pandemic.

In total, 27,718 urine cultures with *E. coli* were reviewed from 2018 to 2021. On average, 6929.5 urine cultures were performed per year. For *E. coli*, the resistance rates to ceftriaxone were 25.21% in 2018, 26.05% in 2019, 24.84% in 2020 and 24.65% in 2021 (Table 1). The resistance rates to meropenem were 0.08% in 2018, 0.13% in 2019, 0.10% in 2020, and 0.30% in 2021 (Table 1). The average *E. coli* with ceftriaxone resistance rates were 25.63% and 24.75% before and after the COVID-19 pandemic, respectively (Fig. 2). The average rates of *E. coli* with CRE were 0.11% and 0.20% before and after the COVID-19 pandemic, respectively (Fig. 3). Between 2020 and 2021, a 200% increase in carbapenem-resistant *E. coli* occurred.

**Table 1.** Ceftriaxone Resistance (Possible ESBL) and Meropenem Resistance (CRE) in *E. coli* Isolates from Urine

Year	Total *E coli* Isolates in Urine	Total *E. coli* With Ceftriaxone Resistance (possible ESBL)	*E. coli* Percentage With Ceftriaxone Resistance (possible ESBL), %	Total *E. coli* With Carbapenem Resistance	*E. coli* Percentage With Carbapenem Resistance, %
2018	5,949	1,500	25.21	5	0.08
2019	5,778	1,505	26.05	8	0.13
2020	6,869	1,706	24.84	7	0.10
2021	9,122	2,249	24.65	28	0.30

Note. ESBL, extended-spectrum β-lactamase; CRE, carbapenem-resistant Enterobacterales.

In total, 2,111 bodily fluid cultures with PSAR were reviewed from 2018 to 2021. On average, 527.75 cultures were performed per year. For PSAR, the resistance rates to ceftazidime were 11.79% in 2018, 13.48% in 2019, 15.19% in 2020 and 15.13% in 2021 (Table 2). The resistance rates to meropenem were 2.12% in 2018, 2.53% in 2019, 2.33% in 2020, and 5.35% in 2021 (Table 2). The average rates of PSAR with ceftazidime resistance in bodily fluid were 12.64% and 15.16% before and after the COVID-19 pandemic, respectively (Fig. 2). The average rates of PSAR with carbapenem resistance were 2.33% and 3.84% before and after the COVID-19 pandemic, respectively (Fig. 3). Between 2020 and 2021, a 130% increase in CR-PSAR occurred.

**Table 2.** Phenotypic Resistance Consistent With ESBL (Ceftazidime Resistance) and CR-PSAR (Meropenem Resistance) in PSAR Isolates From Bodily Fluids

Year	Total PSAR Isolates in Bodily Fluid	Total PSAR With Ceftazidime Resistance (possible ESBL)	PSAR Percentage With Ceftazidime Resistance (possible ESBL), %	Total PSAR With Carbapenem Resistance	PSAR Percentage With Carbapenem Resistance, %
2018	424	50	11.79	9	2.12
2019	512	69	13.48	13	2.53
2020	428	65	15.19	10	2.33
2021	747	113	15.13	40	5.35

Note. ESBL, extended-spectrum β-lactamase; PSAR, *Pseudomonas aeruginosa*; CR-PSAR carbapenem-resistant PSAR.

In conclusion, we detected high levels of resistance in *E. coli* isolated from urine samples and rising resistance to carbapenems in PSAR from bodily fluids. On average, resistance to ceftriaxone was present in 25.19% of *E. coli* isolates from urine each year. These resistance patterns suggest that ESBL is common in the Dominican Republic, at levels comparable to other countries in Latin American.^12–14^ Carbapenem resistance was rare in both *E. coli* and PSAR, averaging 0.15% and 3.08% each year, respectively. However, an increase was seen after the COVID-19 pandemic, particularly in PSAR from bodily fluids. With bodily fluids as a source, this increase may be in more complex patients than those from urine samples and raises the concern of a similar effect in hospitalized patients.

In Latin America, the use of azithromycin for COVID-19 was widespread.^10,15^ Antibiotic use increased in hospitalized and ambulatory patients alike, despite efforts from professional societies to discourage their use.^8–10^ Reports showing an increase in AMR in Latin America after the pandemic are emerging.^16^ This finding mirrors a rise of AMR in the United States, where nosocomial infections by ESBL increased by 32%, CRE increased by 35% and AMR PSAR increased by 132% in the first year of the pandemic.^11^

Excess antibiotic use is a significant problem in LMICs.^4,5^ In the Dominican Republic, antimicrobials can be purchased without prescriptions and are available in pharmacies and stores.^6^ Aminopenicillins are commonly used and may contribute to high rates of ESBL. Studies have reported ceftriaxone resistance in >24% of *E. coli* at a pediatric hospital and 46% in hospitalized adults.^17,18^ Circulating ESBL genotypes in the Dominican Republic include *blaCTX* and *blaTEM*.^19^ Future ASP interventions must focus on both ambulatory and hospital settings. Treatment guidelines reflecting local susceptibilities reported herein can be an important patient safety and stewardship tool.

A strength of our study was the large number of samples and wide geographic representation of samples in the Dominican Republic. These samples may provide a valuable portrait of susceptibilities in these communities. The study also had several limitations. All cultures performed during the study period were included; thus, patients with recurrent infections may be overrepresented. The large number of isolates in the study may balance this weakness. Inferring resistance based on phenotypic resistance patterns rather than genotypes is the main limitation of our study. Ceftriaxone nonsusceptibility without ESBL genes can occur. MIC accuracy can be affected by bacterial inoculum, antimicrobial dilutions, and differing enzyme expression.^20^ High inoculum can occur in severe infections and may have an impact on MICs. Ceftazidime resistance may indicate ESBL in PSAR. However, genes other than ESBL may also contribute to ceftazidime nonsusceptibility in PSAR. The lack of patient specific information, such as comorbidities or prior antibiotic use, is another weakness in our study. Despite these weaknesses, we have reported susceptibilities using the same methodology used in routine clinical practice and may provide valuable information for empiric antimicrobial selection and the development of local treatment guidelines.
